# iPhos: a toolkit to streamline the alkaline phosphatase-assisted comprehensive LC-MS phosphoproteome investigation

**DOI:** 10.1186/1471-2105-15-S16-S10

**Published:** 2014-12-08

**Authors:** Tzu-Hsien Yang, Hong-Tsun Chang, Eric SL Hsiao, Juo-Ling Sun, Chung-Ching Wang, Hsin-Yi Wu, Pao-Chi Liao, Wei-Sheng Wu

**Affiliations:** 1 Department of Electrical Engineering, National Cheng Kung University, Tainan, Taiwan; 2 Department of Environmental and Occupational Health, National Cheng Kung University, Tainan, Taiwan; 3 Institute of Chemistry, Academia Sinica, Taipei, Taiwan

**Keywords:** phosphorylation, iPhos, mass spectrometry, phosphoproteome, targeted LC-MS/MS, label-free quantitative proteomics analysis

## Abstract

**Background:**

Comprehensive characterization of the phosphoproteome in living cells is critical in signal transduction research. But the low abundance of phosphopeptides among the total proteome in cells remains an obstacle in mass spectrometry-based proteomic analysis. To provide a solution, an alternative analytic strategy to confidently identify phosphorylated peptides by using the alkaline phosphatase (AP) treatment combined with high-resolution mass spectrometry was provided. While the process is applicable, the key integration along the pipeline was mostly done by tedious manual work.

**Results:**

We developed a software toolkit, iPhos, to facilitate and streamline the work-flow of AP-assisted phosphoproteome characterization. The iPhos tookit includes one assister and three modules. The iPhos Peak Extraction Assister automates the batch mode peak extraction for multiple liquid chromatography mass spectrometry (LC-MS) runs. iPhos Module-1 can process the peak lists extracted from the LC-MS analyses derived from the original and dephosphorylated samples to mine out potential phosphorylated peptide signals based on mass shift caused by the loss of some multiples of phosphate groups. And iPhos Module-2 provides customized inclusion lists with peak retention time windows for subsequent targeted LC-MS/MS experiments. Finally, iPhos Module-3 facilitates to link the peptide identifications from protein search engines to the quantification results from pattern-based label-free quantification tools. We further demonstrated the utility of the iPhos toolkit on the data of human metastatic lung cancer cells (CL1-5).

**Conclusions:**

In the comparison study of the control group of CL1-5 cell lysates and the treatment group of dasatinib-treated CL1-5 cell lysates, we demonstrated the applicability of the iPhos toolkit and reported the experimental results based on the iPhos-facilitated phosphoproteome investigation. And further, we also compared the strategy with pure DDA-based LC-MS/MS phosphoproteome investigation. The results of iPhos-facilitated targeted LC-MS/MS analysis convey more thorough and confident phosphopeptide identification than the results of pure DDA-based analysis.

## Background

Phosphorylation is a crucial protein post-translational modification (PTM) in many biological processes [1]. And many human diseases, such as cancer and the Alzheimer's disease, are discovered to be triggered by the dysregulation of phosphorylation and dephosphorylation [2,3]. In eukaryotes, protein kinases catalyze the addition of phosphate groups to the side chains of hydroxyl-containing amino acids (serine, threonine and tyrosine) [2,3]. In particular, over 45 protein tyrosine kinases have been implicated in the pathogenesis of human cancers [4]. And nowadays tyrosine-phosphorylated (pTyr) proteins are specific targets for the development of potential biomarkers in prognosis, diagnosis and prediction of drug responses [5]. But the low stoichiometry of phosphorylated proteins is still a significant challenge for identifying them [6].

In the past, protein phosphorylation is detected by antibodies recognizing specific phosphorylated epitopes and/or by the use of ^32^P labelled γATP to incorporate labelled phosphorylation into proteins. These labour-intensive procedures are prone to false phosphorylation site assignment and hence are not suitable for comprehensive analysis [7]. With the development of liquid chromatography mass spectrometry (LC-MS) technology coupled with refined protein enrichment methods, such as immobilized metal affinity chromatography with Fe(III) or Ga(III) [8,9], metal oxide affinity chromatography with TiO_2 _or ZrO_2 _[10,11] or phosphoramidate chemistry [12], it is becoming more and more available for large scale phosphoproteome investigation [13,14].

For many years, collision induced dissociation (CID) is the major core method for tandem MS scan of peptides and proteins with/without PTMs. Other fragmentation methods such as electron transfer dissociation (ETC) were also proposed to identify phosphorylated proteins but are generally not efficient to be used in the analysis of lowly-abundant pTyr peptides [15]. When undergoing CID, neutral loss of 98 Da (H_3_PO_4_) for peptides with phosphorylated-serine (pSer) and phosphorylated-threonine (pThr) would occur and impede the identification of these peptides in data dependent MS/MS [16]. Instead, neutral loss scan, such as MS_3 _and MultiStage Activation, that imposes additional activation events on the neutral loss peaks can be utilized to detect pSer and pThr peptides [17,18]. But tyrosine phosphorylation is largely exempt from the β-eliminated neutral loss of 98 Da and are not suitable for these type of neutral loss scan analysis [16]. The identification of pTyr proteins are mainly through the data-dependent acquisition (DDA)-based LC-MS/MS, which adopts some user-defined criteria to serially select the top intense ions in a survey MS scan of all precursor ions for subsequent CID fragmentation and generates the corresponding product ion spectra for protein database searching or manual interpretation. An alternative for detecting tyrosine phosphorylated peptides is to detect selectively the signal at m/z of 216.043 (the immonium ion) [16,19,20]. Though the neutral loss scan experiments, pure DDA-analysis and selective detection of immoniun ion work well in semi-complex mixtures, in real complex samples such as cell lysates these types of analysis tend to fail to identify most of the phosphopeptides due to the low abundance of phosphorylated peptides and the suppression effect in the presence of those non-phosphorylated peptides [21,22].

To overcome this obstacle, there is a proposed alternative in which biologist combine the alkaline phosphatase (AP) treatment to facilitate a reference-based signal data mining for phosphoproteome analysis [23]. In this additional AP treatment after standard phosphopeptide enrichment procedures such as TiO_2 _microcolumns, researchers further focused on the signals with mass shift of some multiples of 79.966 Da (PO_3_^3-^) detected by comparing the phosphopeptide signals and the corresponding dephosphorylated peptide signals. This has been shown to provide more confident and comprehensive phosphoproteome investigation with lower false positive rate [7,24-26]. And this AP-treatment alternative approach targeting on those signals with mass shift are also shown to provide better discrimination of pTyr peptides in the lung cancer cell lysates [27]. While the overall process, including tyrosine-specific antibody treatment, AP treatment, mass shift calculation and label-free quantification, is applicable, the key integration along the pipeline was mostly done by tedious manual work or private in-house software. To promote and facilitate this comprehensive data mining-based phosphoproteome investigation, developing the fully facilitating toolkit is urgent.

To facilitate the overall workflow of the AP-assisted comprehensive phosphoproteome investigation, we developed the software package, iPhos. iPhos is a collection of signal data mining and analysis facilitating tools to streamline the workflow for routine phosphoproteome characterization. The iPhos toolkit includes one assister and three modules. iPhos Module-1 is used for signal processing of the LC-MS signal peaks extracted by peak extraction computational tools to mine out signals with mass shift between the original and dephosphorylated samples. In addition to iPhos Module-1, we further design the iPhos Peak Extraction Assister to help users automate the batch mode msInspect-based peak extraction procedure [28]. iPhos Module-2 provides customized m/z inclusion lists with peak retention time windows for subsequent targeted LC-MS/MS experiments. iPhos Module-3 is designed to aid the pattern-based label-free quantification. Since most of the pattern-based label-free tools did not link the information of peptide identification to the quantification results [29], we developed iPhos Module-3 to connect specific phosphopeptide identifications with quantification results generated by pattern-based label-free quantification tools. In addition, iPhos Module-3 can help researchers filter out those peptides with proper phosphorylation site assignment. The iPhos toolkit aims to facilitate the AP-assisted and data mining-based comprehensive phosphosproteome investigation and quantification. The utility of the iPhos toolkit was evaluated through the analysis of the differential expression levels of pTyr proteins between metastatic lung cancer cells with tyrosine kinase inhibitor-treatment and cells without treatment. We reported the results of the tyrosine phosphosproteome investigation facilitated by iPhos and showed the improved pTyr peptide identification over the results generated by the method of pure DDA LC-MS/MS phosphoproteome analysis. The iPhos toolkit is available online at http://cosbi3.ee.ncku.edu.tw/iPhos/.

## Implementation

**Figure 1:**
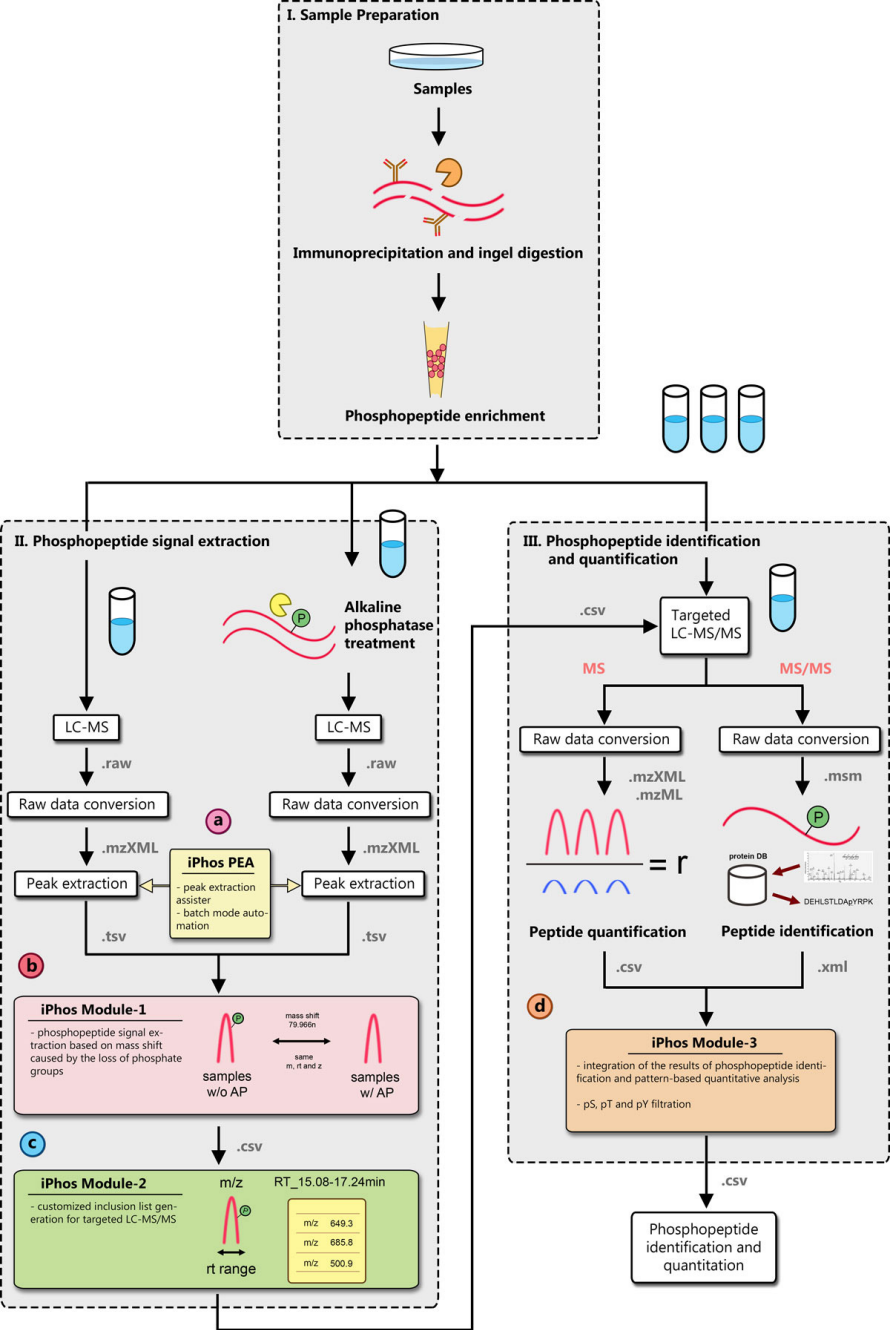
**Overall workflow for the iPhos-facilitated comprehensive phosphoproteome investigation**. This figure is the sketch plot of the overall iPhos-facilitated phosphorylated peptide identification and quantification work-flow. The input and output file formats for different modules are shown in grey. The key integrative tools, collectively called iPhos, to facilitate the workflow are highlighted in (a)-(d). (a) iPhos Peak Extraction Assister automates the msInspect-based batch mode peak extraction. (b) iPhos Module-1 processes the LC-MS data for phosphopeptide signal extraction. (c) iPhos Module-2 generates customized inclusion lists for subsequent targeted LS-MS/MS analysis. (d) iPhos Module-3 links the results of phosphopeptide identification with pattern-based label-free quantitative analysis.

The developed iPhos software toolkit was written in Python 2.6 and the web service was built using the PHP CodeIgniter framework. The overall experiment work-flow of conducting comparison studies based on the AP-assisted phosphoproteome investigation was proposed by various researchers [22-24,26] and was summarized by Wu *et al*. [27] (Figure 1). We developed the key integrative tools, collectively called iPhos, to facilitate the workflow. These key integrative tools are described as follows.

### Raw data conversion

The raw files for the AP-treated and untreated samples generated by mass spectrometers should first be converted into the open file representation format of mzXML, which is now a routine job for analysing MS data. It can be done by suitable programs developed by the Seattle Proteome Centre (SPC) [30]. For example, the ReAdW program can be used for raw files from the LTQ-Orbitrap Velos hybrid mass spectrometers. Or users can use the recently updated tool MSConvert provided in the ProteoWizard project [31], by which a GUI interface was constructed for Microsoft Windows users.

### iPhos peak extraction assister (iPhos PEA)

To perform the signal data mining for extracting phosphopeptide signal, we need first to extract the peak signals from the raw experimental LC-MS data. LC-MS data provides 2-dimensional aspect of the proteome under investigation in retention time and m/z values and the signals forming peaks in this 2-dimensional map represents the potential peptide signals [28,32]. Various algorithms and tools, such as VIPER [33], SuperHirn [34], Peplist [35], OpenMS [36], ICPD [32], and msInspect [28], have been developed to extract the peak signals. In our previous works, we have demonstrated the use of msInspect in the phosphoproteome investigation [27]. Therefore in the development of the iPhos toolkit, we choose msInspect as the default tool for peak extraction, but users are still freely to select any peak extraction software to generate the peak lists. msInspect acquires peak information after peak detection, monoisotopic peak determination and the calculation of original masses. But msInspect requires the user to allocate proper system memory for the java-based application. Further, batch mode for peak extraction is not possible. Hence in the iPhos toolkit, we developed a peak extraction assister that automate the memory allocation and batch mode peak extraction using the default peak extraction tool msInspect. In this peak extraction assister, users can specify the directory where the mzXML files are located and the peak extraction will be automatically completed by the assister (Figure 1-a).

### iPhos Module-1: phosphopeptide signal extraction

iPhos module-1 uses the extracted peak information to find the potential phosphopeptide signals according to mass shift of some multiples of -79.966 Da upon dephosphorylation introduced by the AP treatment (Figure 1-b). Users are freely to select any peak extraction software as long as they provide the header information of the peak list files that is to be used in iPhos Module-1. Up to six pairs of replicas with and without AP treatment, respectively, can be processed by iPhos Module-1. To avoid the inclusion of noise peak signals, users can set the appropriate cut-off values on the extracted peaks. The intensity threshold can be decided with the aid of the visual charts generated in this module. The cut-off value N can be determined using the greatest slope change in the output chart to select only the top N peaks of phosphorylated and dephosphorylated peptides. Finally, users should specify the mass tolerance and retention time window used for signal mass shift mining. In addition, users can also custom the quantity of the mass shift, making iPhos vulnerable to the investigation of other PTMs.

### iPhos Module-2: inclusion list generation for targeted LS-MS/MS

iPhos module-2 was designed so that users can customize the m/z inclusion lists for the downstream targeted LC-MS/MS analysis (Figure 1-c). By setting the user-defined parameters on peptide elution times, users can generate equally-divided inclusion list segments of phosphopeptide candidates with their retention time windows that best suit the downstream MS/MS spectrometer [27]. With the limits of mass spectrometers, duplicated m/z values resulted from different biological replicas will be eliminated based on the round off decimals of m/z values. For comparative experiments, iPhos module-2 can combine signals with mass shifts from samples obtained from different conditions [27].

### iPhos Module-3: integration of phosphopeptide identification and pattern-based label-free quantitative analysis

Since the level of phosphorylation of a protein may be crucial for its function, quantitative information for phosphopeptides is required in the phosphoproteome investigation [17]. While traditional quantification of phosphopreteome is based on labelling methods such as SILAC or iTRAQ [16], the cost-efficient method of label free-quantification method is now a catching-on alternative [26,27,29]. Label-free method can be categorized into three different types [29]. The first type is identification-based quantification using spectral counts. This type of is generally not suitable for phosphoproteome quantification because the identification of phosphorylation is based on only single or few peptide identification [18,24]. The second type is pattern-based quantification using peak identification and XIC calculation. The third type is hybrid-based quantification using the peptide identification as the starting point to calculate the corresponding XIC area. Among these popular quantification methods, the use of SILAC labelling quantification and the pattern-based label-free quantification rely on only MS information. Most of the commercial and open source LC-MS quantification tools based on these two do not link the peptide identification information with the quantification results, thus lacking the phosphorylation site localization.

To facilitate the phosphorylation site localization for quantification tools based on SILAC labelling or pattern-based label-free methods, iPhos Module-3 was designed to integrate the peptide identification from the targeted LC-MS/MS analysis with the quantification results of LC-MS experiments (Figure 1-d). The LC-MS quantification can be done by pattern-based label-free quantification software tools such as msInspect [28] and mzMine [37], which first extract all peaks from each MS data and then align them across different runs [38]. Users are required to input the header information of the quantification results that is to be used in iPhos Module-3. Peptide identification should be the search results by major database search engines such as Mascot. In iPhos Module-3, the properties of peaks, such as m/z, retention time and charge state, are utilized to link the phosphopeptide identification from the targeted LC-MS/MS analyses with the quantification results of the corresponding LC-MS comparative experiments. In addition, in iPhos Module-3 users can specify the phosphorylation type (pSer, pThr, pTyr) in concern to further filter out the intended phosphorylated peptides.

**Figure 2:**
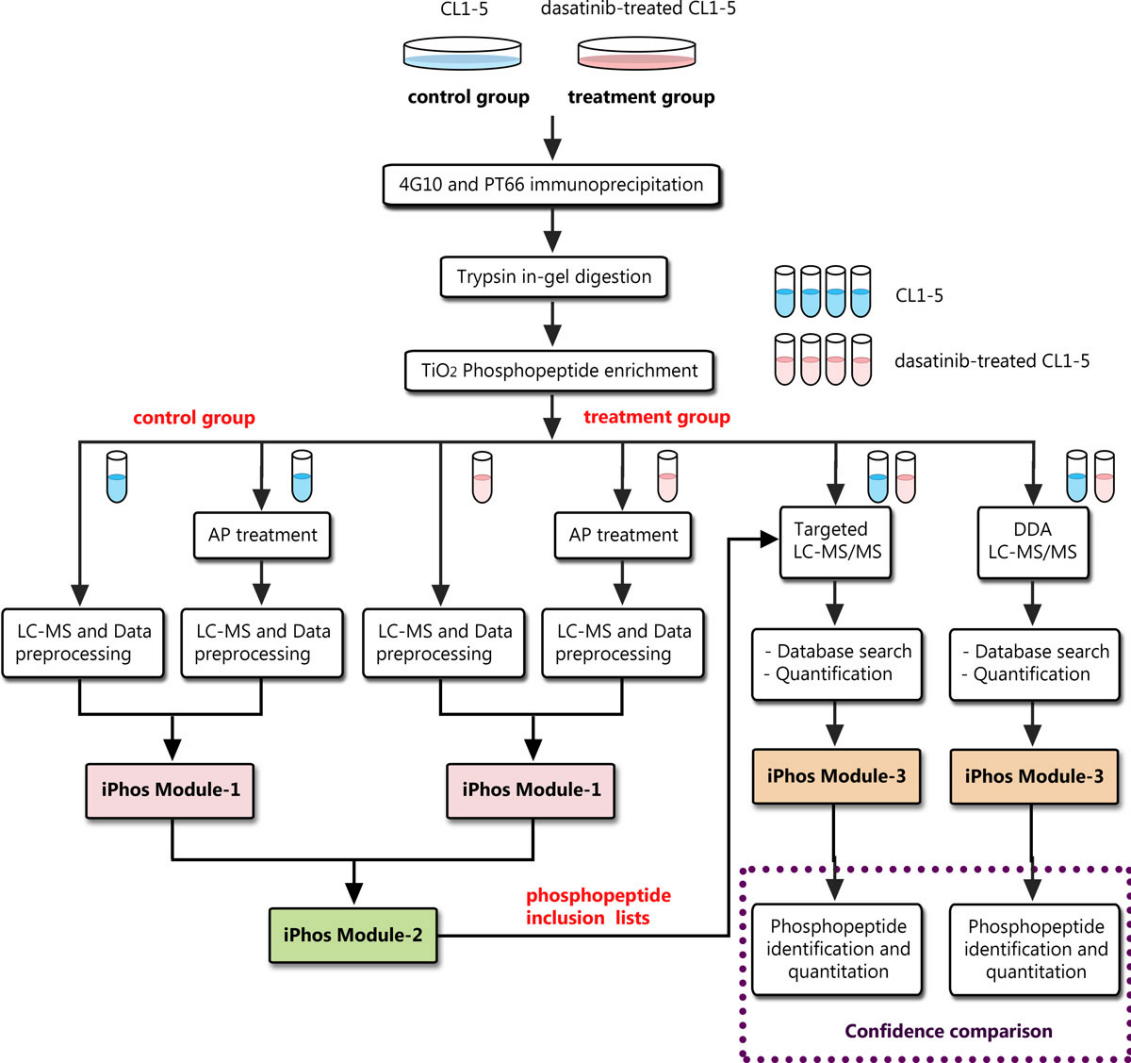
**The experimental settings in this work**. The experimental procedures used in the comparative analysis of the control CL1-5 cells and the dasatinib-treated CL1-5 cells. Beside the analytical strategy of AP-assisted phosphoproteome investigation, we further performed the commonly used strategy of pure DDA LC-MS/MS analysis. For confidence comparison, we assessed the results of the AP-assisted targeted LC-MS/MS and the results of pure DDA LC-MS/MS analysis.

**Figure 3:**
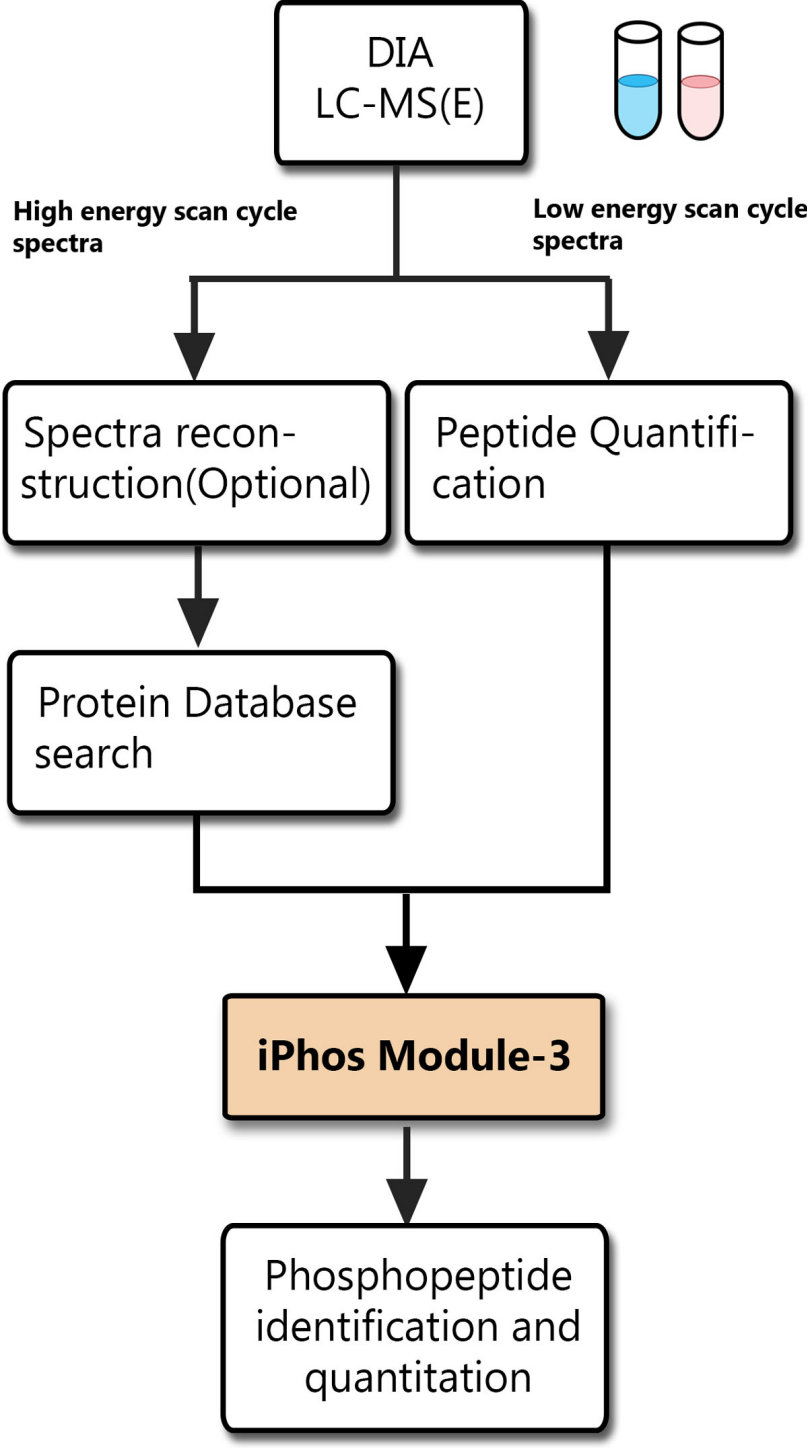
**Extended support for DIA LC-MS methods**. iPhos Module-3 can be extended to support both the DDA phosphoproteome analysis and the DIA phosphoproteome analysis by facilitating the integration of the phosphopeptide quantification results with pattern-based label-free quantification software with the phosphopeptide identification results from the major protein database search engines.

iPhos Module-3 can also facilitate the integration of DDA LC-MS/MS peptide quantification and peptide identification in a similar manner (Figure 2). Moreover, iPhos Module-3 can be extended to support for the catching-on data independent acquisition (DIA) LC-MS methods (Figure 3). In the LC-MS DIA experiment settings, the peptide quantitative information can be acquired for each precursor ion during the low energy scan cycle using standard pattern-based quantification tools [39]. For peptide identification, the composite spectra of MS^E ^data obtained in the elevated energy scan cycle can be analysed with the standard database search tools (such as Mascot) developed for DDA analysis [40], either by directly searching the composite spectra [41] or by searching the reconstructed pseudo-MS/MS DDA spectra [42-45]. This can be done by software products such as ProteinLynx Global SERVER (PLGS), DeMux [42], ETISEQ [45] and other similar tools. To facilitate the DIA phosphopeptide identification, iPhos Module-3 connects the peptide identification results from any of these database search methods with the standard MS peptide quantification results from pattern-based quantification software. And iPhos Module-3 further helps extract those phosphopeptides of interest with Mascot scores above the user-defined threshold.

### Demonstration dataset

#### Cell lysate preparation

The human lung adenocarcinoma cells CL1-5 cells selected by the in-vitro invasion assay were kindly provided by Dr. Pan-Chyr Yang (Academia Sinica, Taipei, Taiwan) and cultured in the RPMI-1640 medium (Gibco, Gaithersburg, MD) supplemented with 10% fetal bovine serum (Gibco), penicillin and streptomycin (Thermo Scientific, Logan, Utah). Cells were incubated at 37°C containing 5% CO_2_. Dasatinib (Santa Cruz Biotechnology, Santa Cruz, CA) was prepared at 0.1 mM in a DMSO vehicle (Sigma-Aldrich, St. Louis, MO). When achieving 70-80% confluence, parts of the cells were treated with a final concentration of 0.1 μM dasatinib in a 0.1% DMSO vehicle for 48 hours. The control and dasatinib-treated CL1-5 cells were centrifuged at 800 × g for 10 min. Both cell pellets were suspended in PBS buffer and subsequently lysed by 2 ml lysis buffer (50 mM Tris-HCl, 150 mM NaCl, 1% NP-40, 0.5% sodium deoxycholate, 0.1% SDS, 1 × protease inhibitor cocktail (Roche Applied Science, Indianapolis, IN), and phosphatase inhibitor mixture (2 mM sodium orthovanadate, 5 mM sodium fluoride, and 10 β-glycerolphosphate)). After centrifuged at 12,000 × g for one hour, the supernatant was collected. Some of the supernatant was then extracted for protein concentration estimation by using a bicinchoninic acid (BCA) protein assay (Pierce, Rockford, IL).

#### Immunoprecipitation and in-gel digestion

1.4 mg of proteins were resuspended in 600 μl of lysis buffer and then reacted with 100 μl of agarose-immobilized pTyr-specific antibody 4G10 (Millipore, Temecula, CA) and PT66 (Sigma-Aldrich). After the antibody treatment, samples were washed three times with 1 ml radioimmunoprecipitation assay (RIPA) buffer and eluted using 80 μl 4× sample buffer (Invitrogen, La Jolla, CA) supplemented with 400 mM dithiothreitol (DTT). To concentrate the eluted proteins, SDS-PAGE composed of a 5% acrylamide stacking gel and a 20% acrylamide separating gel was used. After electrophoresis at 50 V for 30 min, the gel was then stained with Coomassie blue for one hour. The protein band was passed through a destaining solution (H2O/methanol/acetic acid in a ratio of 50/40/10 (v/v/v)) and excised in pieces of 1 mm^3^, which were then subjected to in-gel digestion.

The destained gel pieces were washed twice with solution of 50% (v/v) acetonitrile (ACN) / 25 mM ammonium bicarbonate. The spots were reduced with 10 mM DTT for one hour at 56°C and alkylated with 55 mM iodoacetamide for 45 min in the dark at room temperature. Subsequent digestion was performed by incubating the samples with 0.1 μg of TPCK-treated modified porcine trypsin (Promega, Madison, WI) at 37°C overnight. The extracts of the pieces were prepared using 20 μl of 50% CAN / 5% formic acid; these extracts were combined with the supernatant containing the tryptic peptides. The samples were then dried using a centrifugal evaporation instrument (Vacufuge, Eppendorf, Hamburg, Germany).

#### Phosphopeptide enrichment by TiO_2 _pipette tips and AP treatment

The tryptic peptides were suspended with 100 μl solution A (0.1% Trifluoroacetic acid (TFA), 80% ACN with 300 mg/ml lactic acid) and loaded onto the TiO_2 _pipette tip (200 μ l/3 mg, GL science, Torrance, CA) for phosphopeptide binding. After centrifuge, we repeated the binding step twice. The wash step was performed with solution A and solution B (0.1% TFA, 80% ACN) successively. Then 50 μl 0.5% ammonium hydroxide was added to the tips for elution. A quarter of the eluted samples were further treated with alkaline phosphatase (AP). This quarter of the samples for AP treatment was adjusted with dephosphorylation buffer (Roche) to pH 8-9 and then incubated with 1 U of AP (Roche) at 37°C for 2 h. After that, 2.5% TFA were added to all the eluted samples and the samples were desalted by C18 pipet tips (Varian, Lake Forest, CA). The eluate was evaporated and suspended with 10 μl of 5% ACN in 0.1% TFA solution before nanoLC-MS analysis. For generating the targeted phosphopeptide signals in this study, we categorized four experimental groups: CL1-5, CL1-5 with AP treatment, dasatinib-treated CL1-5, and dasatinib-treated CL1-5 with AP treatment.

#### NanoLC-MS analysis

The samples were analysed by full-scan LC-MS on a nano-ACQUITY system (Waters, Milford, MA) coupled with an LTQ-Orbitrap Velos hybrid mass spectrometer (Thermo Scientific) which is equipped with a PicoView nanospray interface (New Objective). Peptide mixtures were loaded onto a 75-μm × 250-mm nano-ACQUITY UPLC BEH130 column packed with C18 resin (Waters) and were separated at a flow rate of 300 nl/min using a linear gradient of 5% to 40% solvent B (95% acetonitrile with 0.1% formic acid) for 60 min, followed by a sharp increase to 85% solvent B at 1 min and then a holding period at 85% solvent B for another 10 min. Solvent A was 0.1% formic acid in water. The effluent from the HPLC column was directly electrosprayed into the mass spectrometer. The LTQ-Orbitrap Velos instrument was operated in full scan MS acquisition mode. Full scan MS spectra (m/z 300-2000) were acquired in the Orbitrap analyser with resolution set at 60,000 (all Orbitrap system resolution values are given at m/z 400). The standard mass spectrometric conditions for all experiments were as the following: spray voltage, 1.8 kV; no sheath and auxiliary gas flow; heated capillary temperature, 200 °C; predictive automatic gain control (AGC) enabled; and an S-lens RF level of 50%. The instrument was controlled by Tune 2.6.0 and Xcalibur 2.1 software.

#### Targeted LC-MS/MS analysis and database search

The m/z values with retention time windows of possible pTyr peptide signals were obtained from iPhos Module-2. We further divided the remaining half of the samples into two portions. The inclusion lists on the same LTQ-Orbitrap Velos hybrid mass spectrometer triggered MS/MS analysis once these m/z values were captured on one portion of the pooled control and treated samples. The other portion was subjected to data-dependent acquisition (DDA) as a comparison with the targeted LC-MS/MS analysis. Each scan of the mass spectrometer contained one full scan mass spectrum (m/z 300-2000) followed by 20 tandem mass spectra with the collision energy set at 35%. The targeted LC-MS/MS mode was devised that ten most intense ions from the inclusion list were used for LC-MS/MS analysis. In the targeted MS/MS mode, if there was no signal matching the inclusion list in full scan, the DDA mode was used. The method of multi-stage activation was applied and the neutral losses were set as 97.98, 79.97, 48.99, 39.98, 32.66, and 26.66.

All MS raw data were processed by Raw2MSM and searched against the IPI human database (Version 3.87) using an in-house Mascot server (Version 2.2, Matrix Science Ltd., London, UK) with a confidence level of 95%. Up to two missed cleavages were allowed for the trypsin digestion simulation. We allowed the variable modifications of Carboxyamidomethylation on Cys, deamidation on Asp and Glu, oxidation on Met, and phosphorylation on Ser, Thr, and Tyr. Mass deviations for precursor ions and fragment ions were all set to 1 Da. A false discovery rate of ≤ 5% was set based on the calculation by the Mascot-integrated decoy database search.

## Results and discussion

### CL1-5 cell lysate phosphoproteome investigation assisted by iPhos

To demonstrate the utility of the iPhos toolkit in a large-scale quantitative tyrosine phosphoproteomics study, two enriched pTyr protein mixtures were prepared from the control and dasatinib-treated CL1-5 cells. It has been shown that several pTyr proteins involved in Src kinase regulation have different expression levels in the human metastatic lung cancer cell line CL1-5 compared with the less invasive sub-population of CL1-0 [27]. And dasatinib is an anti-tumour drug that inhibits Src family tyrosine kinases and is frequently used to evaluate changes in pTyr protein expression levels before and after treatment [46,47]. pTyr proteins were immunoprecipitated using a combination of two pTyr specific antibodies, 4G10 and PT66. To form a comparison study, we use the CL1-5 cells as the control group and dasatinib-treated CL1-5 cells as the treatment group. Half of both samples were further divided into two portions and one of this sub-portion was treated with alkaline phosphatase. The other half of both samples was also divided into two portions: one portion used for targeted LC-MS/MS analysis and the other portion used for DDA LC-MS/MS analysis. We scattered each part into three technical replicas to measure reproducibility. The overall datasets and settings of the comparison study are shown in Figure 2.

**Figure 4:**
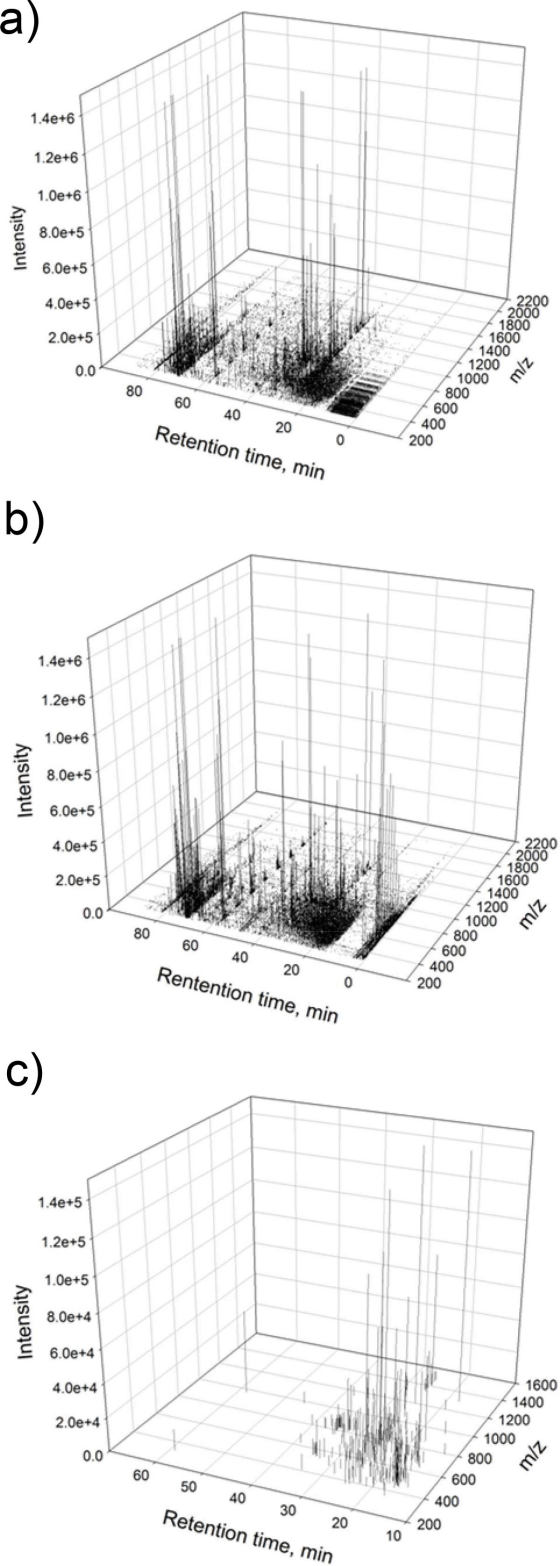
**Illustration of phosphopeptide signal data mining**. Three-dimensional (retention time, m/z and signal intensity) representation of the LC-MS signals before and after the signal data mining of iPhos Module-1. (a) Approximately 19,000 monoisotopic signals are in the tryptic pTyr-enriched protein mixture samples that were not alkaline phosphatase treated. (b) The signals in the tryptic pTyr-enriched protein mixture that has undergone alkaline phosphatase dephosphorylation. (c) iPhos Module-1 extracted 262 possible pTyr signals with mass shifts of some multiples of phosphate group loss.

We set the following parameters in iPhos Module-1: top 3000 peaks cut-off value in phosphorylated and dephosphorylated runs; 79.966 Da mass shift; 0.05 Da mass tolerance; 5 min peak retention time shift tolerance; up to 5 phosphate groups modified; charge state at least 2. Then we loaded the potential phosphopeptide signals mined out from both the control and treatment group by iPhos Module-1 into iPhos Module-2. In iPhos Module-2, we set the time range to be 10 to 60 min according to the exact peak elution time in the gradient of solvent B and generated the inclusion lists by separating these potential pTyr peptides signals into 10 segments with 1.5 min extra retention time tolerance in each segment. The round-off decimal was set to be 2. Overall, 262 unique signals with specific retention time window was found and input as the "inclusion list" in the LTQ-Orbitrap Velos mass spectrometer for targeted LC-MS/MS analysis. These 262 signals were extracted from about 19,000 monoisotopic peaks presented in all LC-MS runs, including the original samples and the dephosphorylated samples (Figure 4).

Then we used iPhos module-3 to combine the quantification results from Progenesis (Nonlinear Dynamics, Newcastle upon Tyne, UK) and the pTyr peptide identification results from the Mascot search engine. The parameters used in iPhos Module-3 was set as the following: Mascot score higher than 25; 0.2 Da m/z tolerance; 2 min retention time tolerance; pTyr peptide modification. Beside the spiked-in internal standard peptides (MSYNCCSGNFSSR), a total of 125 pTyr peptides are identified and quantifiable in the control and treatment group (See Additional File 1). And there are 18 differentially expressed pTyr peptides (using the ANOVA test with α = 0.05, shown in Table 1).

**Table 1:** Eighteen pTyr peptides with differential expression levels between the control and dasatinib-treated CL1-5 cells

	IPI number	Gene Symbol	Protein name	Sequence	m/z	Ratio (control / treatment)	ANOVA
01	IPI00977158	CTSF	Cathepsin F	p**T**RTIp**Y**L**N**TLLRK	826.8552	infinity	3.8E-4

02	IPI00003384	CELSR1	Cadherin EGF LAG seven-pass G-type receptor 1	DRDA**N**SVITp**Y**QLp**T**GG**N**TR	714.9915	8.57	1.7E-2

03	IPI00908866	MGAT4A	Alpha-1,3-mannosyl-glycoprotein 4-beta-N-acetylglucosaminyl-transferase A	Np**Y**SITVp**S**IV**M**GIPTVK	632.9769	4.51	4.4E-2

04	IPI00328929	ZC3H18	Zinc finger CCCH domain-containing protein 18	Rp**T**LSGSGSGSGSSp**Y**SGSSp**S**R	701.9993	3.33	1.0E-2

05	IPI01025392	UBR7	Ubiquitin protein ligase E3 component n-recognin 7	PLA**C**Sp**Y**E**C**HGSHK	542.5891	2.13	3.7E-3

06	IPI01014015	TSPAN19	Tetraspanin 19	p**Y**C**C**G**Q**HNp**Y**p**T**DWIK**N**KNK	805.3546	1.98	2.7E-4

07	PI01009113	POLR2A	cDNA FLJ53860, highly similar to DNA-directed RNA polymerase II largest subunit	TL**N**p**T**FHp**Y**AGVSAK	523.5813	1.65	3.1E-2

08	IPI00025468	SKIL	Ski-like protein	p**Y**LGp**T**PEEKK	612.7616	1.54	3.6E-2

09	IPI00981378	CAMSAP2	Calmodulin regulated spectrin-associated protein family, member 2	p**T**p**S**SVASGTEp**Y**TGPK	812.3648	0.70	2.0E-2

10	IPI00977861	ABI3BP	ABI family, member 3 (NESH) binding protein	p**Y**KNEp**T**LALPAESK	812.3654	0.70	2.0E-2

11	IPI00853187	LILRB3	Leukocyte immunoglobulin-like receptor subfamily B member 3	CGp**SQ**KGp**Y**HHFVL**M**KE	970.9699	0.67	3.1E-2

12	PI00878191	DRG1	Developmentally-regulated GTP-binding protein 1	p**Y**LLEKIWDp**Y**LK	822.3957	0.35	2.2E-2

13	IPI00738852	RBM44	RNA-binding protein 44	ILGEp**Y**p**T**SPLp**S**SK	767.3362	0.34	3.2E-2

14	IPI00747652	cDNA FLJ60027	Moderately similar to F-box only protein 25	RHGp**Y**Cp**T**LGETF**N**R	857.407	0.17	3.1E-2

15	IPI00743720	SLC25A37	Mitoferrin-1	p**T**Vp**YQ**LNGLAGp**Y**FK	857.4067	0.17	3.1E-2

16	IPI00001617	SOX4	Transcription factor SOX-4	Sp**S**AASSPAAGRSPADHRGp**Y**Ap**S**LR	837.7143	3.2E-2	2.7E-2

17	IPI00016594	C2orf42	Uncharacterized protein C2orf42	p**S**FIQNRDGTp**Y**ELFK	938.9226	1.3E-2	1.8E-2

18	IPI00382739	DHX34	Probable ATP-dependent RNA helicase DHX34	p**Y**RINLSVLGPAp**T**R	810.8598	0.3	2.1E-2

### Demonstration example of AP-assisted phosphopeptide identification

**Figure 5:**
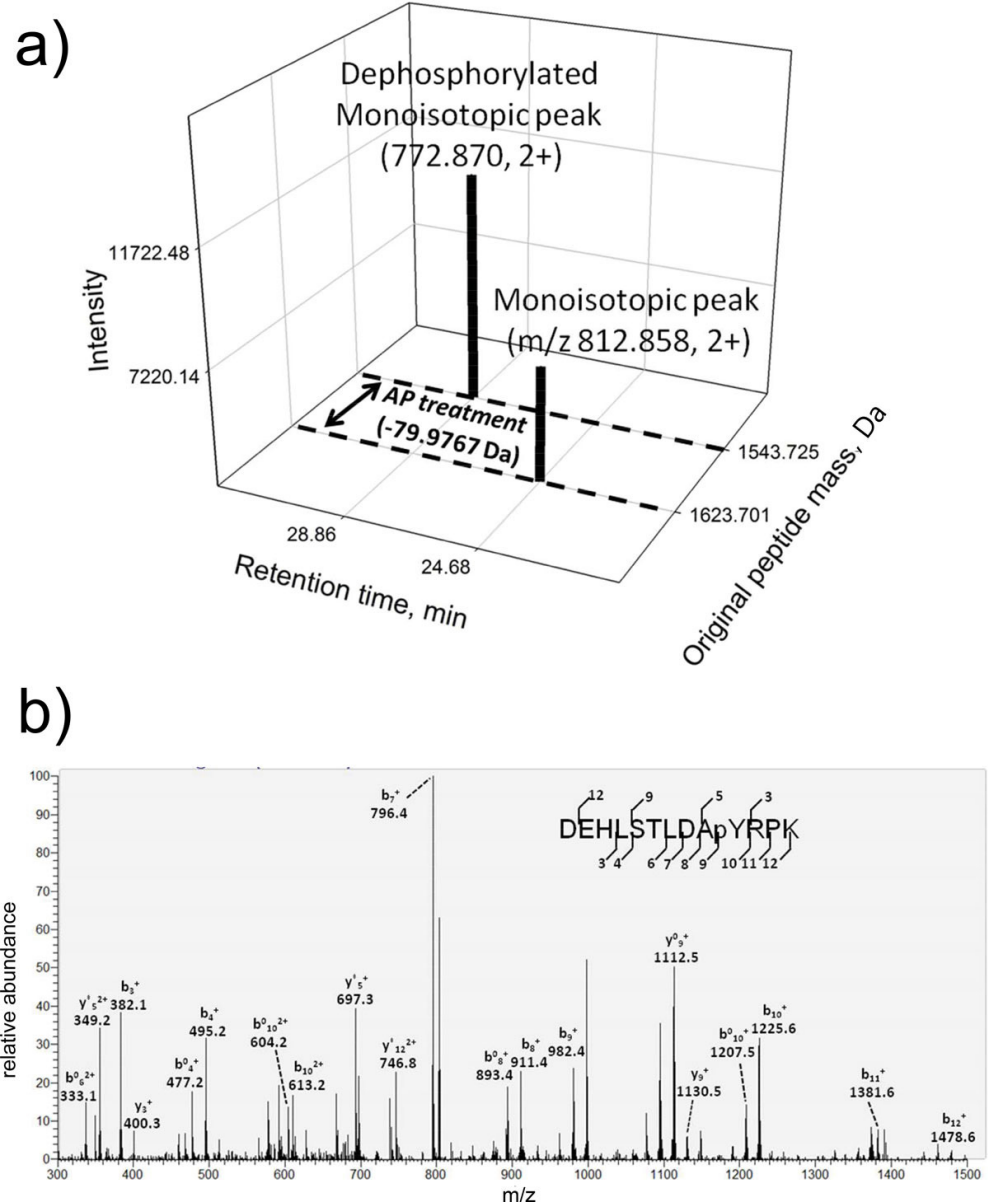
**MS/MS spectra of the peptide DEHLSTLDApYRPK**. This is a demonstrating example of the iPhos-facilitated phosphopeptide investigation. (a) From iPhos Module-1 and Module-2, the monoisotopic peak with m/z value of 812.858 was selected for targeted LC-MS/MS analysis. (b) The MS/MS spectrum of the identified pTyr peptide DEHLSTLDApYRPK. This peptide was identified to be the fragment of the protein "Similar to ankyrin repeat domain 20A, isoform CRA_a" (m/z 812.86, 2+) with a Mascot score of 39.

The central dogma of the iPhos-facilitated phosphoproteome investigation is based on the loss of phosphate group(s) (-79.966n Da) to extract potential phosphopeptide signals as the inclusion lists for targeted LC-MS/MS analysis. We take a demonstration example from our datasets to show the idea. The partial sequence (DEHLSTLDApYRPK) of the protein named "Similar to ankyrin repeat domain 20A, isoform CRA_a" was identified in all targeted LC-MS/MS runs. After signal data mining of iPhos Module-1 and Module-2, the original peptide mass of 1623.701 Da (from m/z 812.858, 2+) in the AP-untreated run compared with 1543.725 Da (from 772.870, 2+) in the AP-treated run reveals a mass shift of -79.9767 Da (HPO_3_) (Figure 5-a). The m/z of 812.8579 was rounded to 812.86 and set as the potential peak signal in the inclusion list with retention time window from 21.51 to 28.43 min. After the targeted LC-MS/MS scan, the pTyr peptide sequence of DEHLSTLDApYRPK was identified and reported by the Mascot search engine with a score of 39 and eluted in the time range of 23.32 to 27.25 min. The MS/MS spectrum of this peptide signal is shown in Figure 5-b. Notice that the elution time found by the MS/MS analysis coincided with the retention time window provided by iPhos Module-2.

### Improvement over DDA-based phosphoproteome investigation

#### iPhos-facilitated investigation obtains more pTyr peptide identification and reproducibility

We have divided our samples to separately perform the iPhos-facilitated target LC-MS/MS analysis and DDA LC-MS/MS analysis for comparison. These two sub-portions are identically prepared but are subject to different LC-MS/MS analysis. We manually collected the identified pTyr peptides in these two analyses. There are 308 and 255 pTyr peptides identified by the Mascot search engine (score ≥ 25) in the targeted LC-MS/MS analysis and DDA LC-MS/MS runs, receptively. A 20.1% improvement on the number of identified pTyr peptides was obtained from the iPhos-facilitated phosphoproteome investigation. Of these identified pTyr peptides, 117 pTyr peptides identified in the targeted LC-MS/MS analysis were found in more than one technical replicas and showed reproducibility. In comparison, only 66 pTyr peptides identified in the DDA LC-MS/MS analysis embraced reproducibility. These results are in agreement with observations in other recent studies [48-50].

#### Confidence evaluation

Owing to the presence of multiple potential phosphorylation sites in a peptide, the common scoring system in peptide search engines such as Mascot and Sequest do not properly evaluate phosphorylation site assignment [51-53]. Two solutions were proposed. One is to use the Mascot Delta Score (MD-score) for proper evaluation of the phosphorylation site localization [53]. The MD-score method calculates the difference between the top two Mascot ion scores of alternative phosphorylation sites for the identical peptide sequence from Mascot search results. The other evaluation metric is provided by PhosphoRS, a tool to automate confident localization of phosphorylation sites [54]. PhosphoRS provides the individual probability values for each putative phosphorylated site based on the given MS/MS data. We used both the MD-score and PhosphoRS (Proteome Discoverer: version 1.3, Thermo. Scientific) to compare the confidence of the uniquely identified pTyr peptides from the targeted LC-MS/MS analysis and the DDA LC-MS/MS analysis.

**Figure 6:**
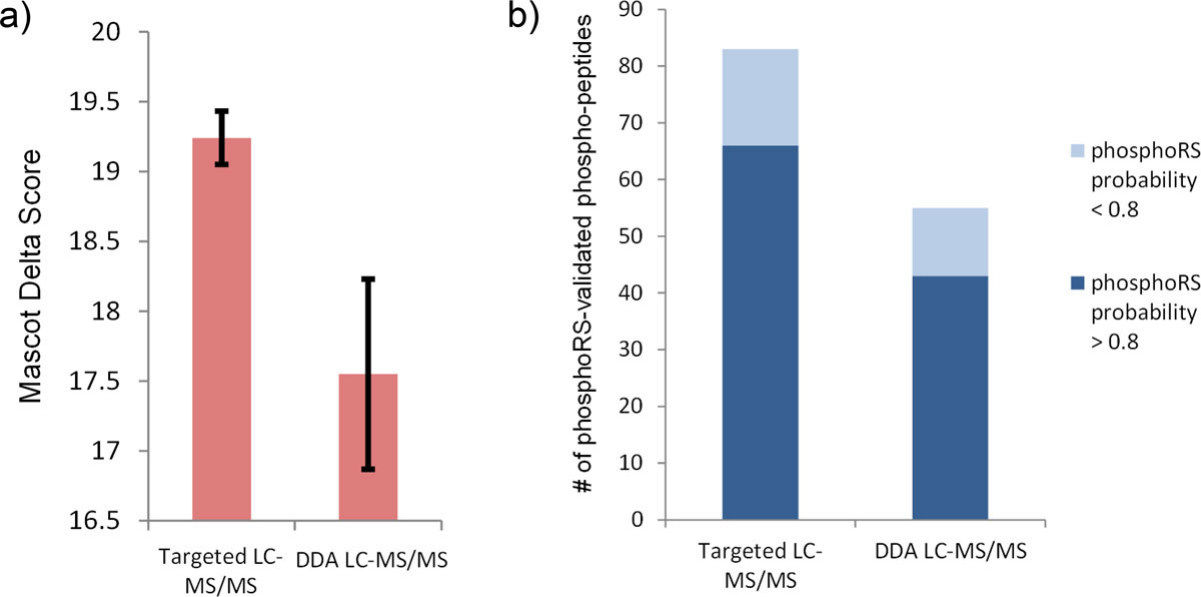
**Confidence evaluation of the targeted LC-MS/MS and DDA-LC-MS/MS phosphoproteome investigation**. Using the MD-score and PhosphoRS methods, we showed the improvement of the targeted LC-MS/MS-based phosphoproteome investigation over DDA-based analysis. (a) The comparison of the assessment results by Mascot Delta Score (MD-score). The scores of the identified pTyr peptides in the targeted LC-MS/MS runs are higher than those identified pTyr peptides in DDA LC-MS/MS runs (19.24 ± 0.19 vs. 17.55 ± 0.68). (b) The comparison of the assessment results by the phosphoRS algorithm in Proteome Discover Software. 83 pTyr peptides (83/191) identified in the targeted LC-MS/MS runs pass the validation. Further, 66 of these validated phosphorylation localization are of high confidence (with probability higher than 0.8). In comparison, only 55 pTyr peptides (55/189) identified in the pure DDA LC-MS/MS runs do. And only 43 of them are of high confidence.

As shown in Figure 6-a, the MD-scores of the identified pTyr peptides are higher in the targeted LC-MS/MS analysis (19.24 ± 0.19) than the MD-scores in DDA LC-MS/MS analysis (17.55 ± 0.68). And it is reported that the MD-score should be more than 5 so as to unambiguously determine phosphorylation sites [55,56]. In our experimental results, only 15.18% (29/191) of the MD-scores in targeted LC-MS/MS analysis are less than 5. In contrast, 24.86% (47/189) of the MD-score in DDA LC-MS/MS analysis does not reach above the threshold of 5. From the evaluation of the MD-score method, iPhos-facilitated AP treatment phosphoproteome investigation demonstrates the improvement in the phosphorylation site localization confidence over conventional investigation using DDA LC-MS/MS analysis.

The evaluation results using the PhosphoRS are shown in Figure 6-b. In the results of the targeted LC-MS/MS analysis, 43% (83/191) phosphorylation localization of the unique pTry peptides are calculated to be confident under the PhosphoRS algorithm. Compared with the results of the targeted LC-MS/MS, only 29% (55/189) are calculated to be confident in the results of the DDA LC-MS/MS analysis. And among these confident phosphorylation localization, 79.51% (66/83) of them in the targeted LC-MS/MS analysis and 78.18% (43/55) in the DDA LC-MS/MS analysis are reported to have probability values of correct phosphorylation localization higher than 0.8, a cut-off value reported to be of acceptable false localization rate. In summary, the evaluation based on the PhosphoRS algorithm shows that iPhos-facilitated AP treatment phosphpoproteome investigation provides more confident phosphorylation localization in comparison with conventional investigation using DDA LC-MS/MS analysis.

## Conclusions

In this study, we developed the iPhos toolkit to facilitate and to streamline the AP-assisted phosphoproteome investigation and evaluated the usability of iPhos through the comprehensive pTyr phosphoproteome investigation of the CL1-5 cells. And we also demonstrated the overall improvement of the iPhos-facilitated phosphoproteome investigation in the thorough phosphopeptide identification and confident phosphorylation localization over the pure DDA-based method. In conclusion, iPhos facilitates the pipeline of AP-based comprehensive phosphopeptide investigation, allowing for rapid production of vital biological researches on protein phosphorylation with higher confidence.

## List of abbreviations

PTM, post-translational modification; pTyr, tyrosine-phosphorylated; LC-MS, liquid chromatography mass spectrometry; pSer, serine-phosphorylated; pThr, threonine-phosphorylated; AP, alkaline phosphatase; DDA, data-dependent acquisition; MD-score, Mascot Delta score.

## Availability and requirements

**Project name: **iPhos

**Project home page: **http://cosbi3.ee.ncku.edu.tw/iPhos/

**Operating system(s): **Windows operating system

**Programming language: **Python 2.6

**Other requirements: **Java SDK

**License: **none

**Any restrictions to use by non-academics: **none.

## Competing interests

The authors declare that they have no competing interests.

## Authors' contributions

PCL and HIW conceived the research topic. HTC, CCW and THY developed the iPhos toolkit and set up the iPhos website. SLH generated the datasets and conducted the LC-MS/MS experiments. JLS and THY analysed the experimental data. THY and SLH wrote the manuscript. PCL and WSW provided essential guidance. All authors have read and approved the final manuscript.

## Supplementary Material

Additional file 1 **The quantitative information of the pTyr peptides identified by the targeted LC-MS/MS analysis in control and dasatinib-treated CL1-5 cells**. Click here for file

## Declarations

The publication of this manuscript was funded by Ministry of Science and Technology of Taiwan (MOST100-2113-M-006-002-MY3).

This article has been published as part of *BMC Bioinformatics *Volume 15 Supplement 16, 2014: Thirteenth International Conference on Bioinformatics (InCoB2014): Bioinformatics. The full contents of the supplement are available online at http://www.biomedcentral.com/bmcbioinformatics/supplements/15/S16.
